# Droplet Size Reduction of Self-Emulsifying Drug Delivery System (SEDDS) Using the Hybrid of Medium and Long-Chain Triglycerides

**DOI:** 10.3390/pharmaceutics17070822

**Published:** 2025-06-25

**Authors:** Kaijie Qian, Yuanyuan Lin, Bingxiang Zhao, Xiangrui Liu

**Affiliations:** 1 Department of Pharmacology and Department of Gastroenterology of the Second Affiliated Hospital, Zhejiang University School of Medicine, Hangzhou 310058, China; l240276@zju.edu.cn (K.Q.); linyuanyuan@zju.edu.cn (Y.L.);; 2 Innovation Center of Yangtze Delta, Zhejiang University, Jiaxing 314100, China; 3 Center for Medical Research and Innovation in Digestive System Tumors, Ministry of Education, Hangzhou 310009, China

**Keywords:** droplet size, self-emulsifying drug delivery system (SEDDS), medium- or long-chain triglyceride, oral bioavailability

## Abstract

**Background:** Self-emulsifying drug delivery system (SEDDS) is widely used to improve the oral bioavailability of hydrophobic drugs. Emulsion droplet size was revealed to be a critical parameter that influences the thermodynamic stability, drug solubility, and drug absorption of the SEDDS. A high proportion of surfactant and/or co-surfactant was usually employed to reduce the particle size, which may lead the low drug loading and undesirable gastrointestinal toxicity. **Methods:** This manuscript proposed a novel strategy to reduce the particle size of emulsions using the hybrid of medium and long-chain triglyceride (MCT and LCT) SEDDS without promoting the concentration of surfactants and co-surfactants. The composition of SEDDS was selected based on the drug solubility. Particle size distribution and zeta potential of emulsion particles were determined using the dynamic light scattering technique. The bioavailability of formulations was evaluated in a mouse model. **Results:** The particle size of the emulsion was reduced from 113.50 ± 0.34 nm (MCT SEDDS) and 371.60 ± 6.90 nm (LCT SEDDS) to 21.23 ± 0.30 nm (MCT&LCT SEDDS). Progesterone, a poorly water-soluble drug, was selected as the model drug in the investigation of SEDDS. The hybrid of MCT&LCT progesterone SEDDS exhibited reduced particle size, enlarged self-emulsifying ranges, and increased drug content in the aqueous phase after lipolysis compared with the conventional mono-MCT or LCT SEDDS. In addition, the bioavailability of progesterone in the MCT&LCT SEDDS formulation was 3.82-fold higher than that of Utrogestan^®^ (a clinical oral administrated product) in a mouse model.

## 1. Introduction

The advent of new drug discovery techniques has significantly accelerated the timeline of drug development pipelines [1,2]. As a result, the number of new lipophilic drug candidates has risen significantly, with approximately 90% of newly synthesized compounds exhibiting aqueous solubility limitations [3]. Consequently, solubility enhancement techniques have become critical in the ease of new drug discovery [4,5]. Among these, lipid-based formulation represents a clinically validated approach to enhance the solubility and bioavailability of poorly water-soluble drugs [6,7,8]. Self-emulsifying drug delivery system (SEDDS) is a typical lipid-based formulation that consists of oil, surfactant, and/or co-surfactant/co-solvent. Due to the successful improvement of drug solubility and bioavailability, numerous commercial products like Neoral^®^, Norvir^®^, and Rapamune^®^ et al. have been approved by the Food and Drug Administration (FDA) in recent decades, using the SEDDS technique.

Particle size is an essential parameter in the design and development of SEDDS products. Based on the emulsion’s droplet size distribution and physical stability, the SEDDS could be classified as (1) self-microemulsifying drug delivery system (SMEDDS), generating transparent microemulsions with 10 to 100 nm droplets; (2) self-nanoemulsifying drug delivery systems (SNEDDS), producing 100–250 nm droplets; and (3) macroemulsifying systems with droplets >1 μm [9,10]. Microemulsions are a thermodynamically stable system, resulting in long-term, transparent solutions with oil-in-water droplets [11,12]. While larger droplets in SNEDDS and macroemulsions exhibit metastability, leading to phase separation or turbidity. Typically, reducing the particle size of the emulsion enables the enhanced interfacial surface of the drug on the intestinal lumen, thus increasing the solubility of hydrophobic drugs. Furthermore, a smaller particle size and optimized zeta potential may facilitate the mucus penetration profile of SEDDS. Griesser et al. suggested that the SEDDS has a size of 25 nm and exhibited larger mucus penetration than those of formulations with sizes ranging from 50 to 500 nm [13].

To achieve these benefits, high surfactant loadings are traditionally required to reduce interfacial tension, minimize droplet size, and enhance stability [14,15,16,17]. In addition, the surfactant and co-surfactant may be added to prevent the emulsified oil droplets from the subsequent drug precipitation, coalescence, and Ostwald ripening [18,19,20,21]. However, excessive surfactant-to-oil ratios (SOR) will undermine drug loading and may bring gastrointestinal toxicity [22]. Therefore, the number of clinical trials of SEDDS has reduced in recent years despite the advantages of this formulation, like thermodynamic stability, small droplet size after emulsifying, higher solubility, and higher gastrointestinal uptake et al. [23]. To address this, we proposed an alternative approach for forming SEDDS without improving the SOR value.

Triglyceride, a type of ester that is derived from glycerol and three fatty acids, was commonly applied as an oil phase in the SEDDS. Triglyceride was always revealed to be a kind of safety pharmaceutical excipient since it was nature-derived and can be digested and absorbed in the intestinal tract [24]. According to the length of fatty acid, the triglycerides can be classified as medium-chain length triglycerides (MCT, e.g., coconut oil and palm seed oil) and long-chain length triglycerides (LCT, e.g., corn oil, soybean oil, olive oil, peanut oil, sesame oil, and sunflower oil) [23]. In recent decades, abundant excellent works paid attention to the fatty acid chain length, degree of saturation, degree of digestion, and the approach of absorption into the system circulation [25,26,27,28,29,30]. Prior studies have extensively characterized either MCT or LCT-based SEDDS. However, rarely work was concerned with the influence of the blended oil phase on the particle size of emulsions. Here, a poorly water-soluble drug, progesterone, was selected as the model molecule. A novel progesterone SEDDS was developed using an MCT&LCT mixed oil phase, achieving a droplet size of around 20 nm and enhanced bioavailability without increasing SOR. This strategy applies the complementary advantages of MCT and LCT, offering a promising approach to the limitations of conventional SEDDS formulations.

## 2. Materials and Methods

### 2.1. Materials

Progesterone (99.4% purity) was provided by China Resources Zizhu Pharmaceutical Co., Ltd. (Beijing, China). Maisine CC (Glycerol/Glyceryl monolinoleate), Peceol (Glyceryl monooleate), Plurol Oleique CC497 (Polyglyceryl-3 dioleate), Labrasol (Caprylocaproyl Polyoxyl-8 glycerides), and Transcutol HP (Diethylene glycol monoethyl ether) were supplied by GATTEFOSSE (Saint-Priest, France). Castor oil was bought from Sinopharm Chemical Reagent Company, Ltd. (Shanghai, China). Caprylic/capric triglyceride was purchased from Lv Sen Chemical Company, Ltd. (Shandong, China). Glyceryl Triacetae was bought from MCE (Monmouth Junction, NJ, USA). Sunflower seed oil was bought from China Oil and Foodstuffs Corporation (Beijing, China). Sesame seed oil was bought from Anhui Fengyang Yushan Oil Company, Ltd. (Fengyang, China). Flax seed oil was provided by Shanghai Yuanye Bio-Technology Company, Ltd. (Shanghai, China). Span 80 (Sorbitan monooleate) was purchased from Biobasic (Toronto, ON, Canada). PEG 400 (Polyethylene glycol 400) was bought from Jiangxi Yipusheng Pharmaceutical Company, Ltd. (Nanchang, China). Tween 80 (polysorbate 80) and Tween 20 (polysorbate 20) were from Sigma-Aldrich (St. Louis, MO, USA). All other chemicals and solvents were reagent grade and used without further purification.

### 2.2. Animals

Male ICR mice (30 ± 2 g) were obtained from Hangzhou Celojin Biotechnology Company, Ltd. (Hangzhou, China) and housed in standard conditions. Animal experiments were approved by the Animal Experimental Ethical Committee of Zhejiang University (ethical approved code: ZJU 20250361).

### 2.3. Drug Concentration Determination

High-performance liquid chromatography (HPLC) was employed to determine the concentration of progesterone in formulations and plasma samples. The HPLC system was equipped with a Primaide 1110 pump, a Primaide 1210 autosampler, a Primaide column oven, a Primade UV detector, and a Phenomenex C18 (250 mm × 4.6 mm, 5 μm) column. The detection wavelength was set at 241 nm. Samples were analyzed using a mobile phase consisting of methanol/water at a ratio of 8:2 (*v*/*v*). The flow rate was set at 1.0 mL/min, and the column temperature was set at 40 °C. Before the determination of progesterone concentration in samples, in vitro and in vivo calibration curves were constructed.

#### 2.3.1. Calibration Curve of in Vitro Samples

A series of standard progesterone methanol solutions with the drug concentration ranging from 1 to 100 μg/mL was prepared to establish a calibration curve. The standard solutions were determined using the HPLC method in Section 2.3. The peak areas of each known concentration sample were used to fit the calibration curve of in vitro samples.

#### 2.3.2. Calibration Curve of Mouse Plasma Samples

The progesterone methanol standard solutions with concentrations of 20, 100, and 200 μg/mL were prepared using the volumetric flasks. A total of 95 μL of mouse plasma was mixed with 5 μL of the methanol standard solutions and vortex-mixed for 1 min. The drug concentration in mouse plasma was 1, 5, and 10 μg/mL. Next, 100 μL of these plasma samples were then mixed with 100 μL saturated ammonium sulfate. The proteins in these plasma samples were removed by adding 400 μL of methanol, followed by vortex-mixing for 5 min. Samples were then centrifuged at 20,200× *g* for 10 min. The supernatants were transferred to a new 1.5 mL tube, and nitrogen was flushed over them at 50 °C until all the solvent evaporated. The samples were redissolved using 100 μL of methanol followed by vortex-mixing for 5 min and centrifugation at 20,200× *g* for 10 min. The supernatants were analyzed using the HPLC method described in Section 2.3. The peak areas of each sample were used to fit the calibration curve of plasma samples.

### 2.4. Formulation Optimization

#### 2.4.1. Determination of Drug Solubility in Excipients

The saturated solubility of progesterone in oils (castor oil, maisine CC, peceol, glyceryl triacetate, caprylic/capric triglyceride, sunflower seed oil), surfactants (cremophor EL, Tween 20, Tween 80), and co-surfactants (Labrasol, PEG 400, Transcutol HP, plurol oleique CC497, Span 80) was determined using the HPLC method. An excessive amount of progesterone was added into a glass vial with 2 mL of excipients. To accelerate the process of solubilization, the supersaturated drug-excipient mixture was placed in a shaking incubator at 25 °C, 100 rpm for 48 h. The sample was then centrifuged at 15,000× *g* for 10 min to eliminate the precipitate. The supernatant liquid was diluted with methanol and the drug concentration in samples was measured by HPLC.

#### 2.4.2. Pseudo-Ternary Phase Diagram Construction

The oil, surfactant, and co-surfactant that exhibited the highest solubility of progesterone were optimized for constructing the SEDDS formulation. Maisine CC (LCT) and Glyceryl Triacetae (MCT) were selected as the oil phase. Tween 80 and Transcutol HP were selected as surfactant and co-surfactant, respectively. At a given weight fraction of surfactant in the system from 40% to 70% (at 5% intervals), the co-surfactant was added into each system at a weight fraction from 0% to 30% (at 5% intervals). The oil phase (weight fraction from 0% to 60%) was added into each system until the overall weight fraction reached 100%. In addition, the influence of the single and blended triglycerides on the formation of SEDDS was also investigated in this work. The oil phase was constructed with pure LCT, pure MCT, and the mixture of MCT and LCT with a weight ratio of 1:1. The mixture of oil, surfactant, and co-surfactant was weighed in a glass beaker and mixed until the homogeneous phase was generated at 25 °C. Moreover, 100 mg mixture was then gradually added into 10 mL Millipore water at 25 °C (with a weight ratio of 1:100). The pseudo-ternary phase diagrams were constructed, where the self-emulsification area was identified as a transparent or semi-transparent solution.

### 2.5. Characterization

#### 2.5.1. Formulation Preparation

Based on the pseudo-ternary phase diagrams, the weight fraction of oil-surfactant-co-surfactant SEDDS formulation was set at 20:55:25. To investigate the influence of oil phase on the emulsion particle size, zeta-potential and lipolysis performances of formulation, MCT-SEDDS, LCT-SEDDS, and MCT&LCT-SEDDS were prepared and characterized. The formulation composition is presented in Table 1. The MCT, LCT, and MCT&LCT-SEDDS formulations were prepared by dissolving 1 g of progesterone in 50 g of oil-surfactant-co-surfactant homogeneous mixture. Transparent and clear solutions were obtained after stirring at 37 °C.

#### 2.5.2. Droplet Size Analysis and Zeta Potential Measurement

The droplet size, zeta potential, and polydispersion index (PDI) of emulsified droplets were measured using a Zetasizer Nano-ZS (Malvern panalytical, Malvern, UK). Samples were prepared by dispersing 100 mg of each SEDDS formulation in 10 mL of Millipore water and filtering through 1 μm filters. In order to investigate the influence of the MCT/LCT ratio on the particle size of emulsions, a series of SEDDSs were prepared with a gradient MCT/LCT ratio in the oil phase (as shown in Table 2). Data were collected over 2 min with 3 replicates for each formulation. The particle sizes are reported as the volume–size distribution.

#### 2.5.3. In Vitro Lipolysis

The lipolysis media was composed of 150 mM NaCl, 50 mM tris maleate, and 50 mM CaCl_2_. The solution of pancreatic lipase enzyme was prepared by suspending 5 g of pancreatin in 5 mL of lipolysis media and homogenized thoroughly by using a magnetic stirrer. The solution was then centrifuged at 4500 rpm for 5 min at 4 °C. The supernatant of the solution was placed in a refrigerator at 4 °C for subsequent use.

Lipolysis experiments were adopted according to the procedure described by Alaadin Alayoubi et al. [31]. The experiment medium comprised 45 mL of lipolysis media and 5 mL of pancreatic lipase enzyme solution. The medium was continuously stirred and equilibrated at 37 °C for 10 min. At the beginning of the test, 250 μL of the MCT-SEDDS, LCT-SEDDS, and MCT&LCT-SEDDS (containing 5 mg of the drug) were added to the experiment medium, which had been pre-titrated to pH 6.5. During the lipolysis process of triglycerides, a pH-titrator unit (T5 Excellence, Mettler Toledo Inc., Columbus, OH, USA) was used to prevent the pH from decreasing due to the generation of free fatty acid.

The automatic titration process was lasted for 30 min and terminated by adding 1 mL of 0.3% p-aminophenyl palmitate, which was composed of 10% isopropyl alcohol and 90% 50 mM Tris buffer (pH = 8.0), followed by incubation at 45 °C for 20 min. The drug distribution after the lipolysis test was studied using the ultracentrifugation method with an Optima XL-100 K Ultracentrifuge (Beckman Coulter, Brea, CA, USA). This is a dynamic approach for separating the drug in the aqueous phase, lipid droplets, or sedimentation. After ultracentrifugation at 360,000× *g*, 4 °C for 1.5 h, three layers could be obtained: (1) a floating layer of undigested lipids; (2) an aqueous layer of re-emulsified components; (3) a layer of precipitated components. The drug concentration in the aqueous phase and the precipitation layer were determined using the HPLC method (Section 2.3.1).

### 2.6. Thermodynamic Models of SEDDS

At a given pressure and temperature, the Gibbs free energy of colloidal formation ($\Delta G_{formation}$) was associated with the interfacial free energy ($\Delta G_{I}$) and a configuration entropy term ($-T\Delta S_{config}$) [32]:

(1) $$\Delta G_{formation}=\Delta G_{I}-T\Delta S_{config}$$

where the interfacial free energy term ($\Delta G_{I}$) could be explained by the change in the contact area between the oil and water phase (ΔA) and the interfacial tension (γ) [33]:

(2) $$\Delta G_{I}=\gamma \Delta A$$

Therefore, the term $\Delta G_{formation}$ could be expressed as

(3) $$\Delta G_{formation}=\gamma \Delta A-T\Delta S_{config}$$

At a given temperature and composition, the change in interfacial free energy ($\Delta G_{I}$) is always positive in the process of the droplet formation since taking the place of colloids increases the contact area (ΔA) between two phases, and the interfacial tension (γ) is always a positive term. Therefore, the term interfacial free energy ($\Delta G_{I}$) is a thermodynamically unfavorable parameter that hinders the process of emulsion particle generation.

The configuration entropy is related to the location of constituent particles [34]. In a well-dispersed SEDDS, it ($-T\Delta S_{config}$) is always a negative term that favors the colloid formation. This term can be rewritten as [32]:

(4) $$\Delta S_{config}=-\frac{nk}{\emptyset }\left(\emptyset ln\emptyset +\left(1-\emptyset \right)\ln\left(1-\emptyset \right)\right)$$

where n is the number of particles, k is the Boltzmann’s constant parameter, ∅ is the volume fraction of the oil phase. The free energy of colloidal formation can then be described using the equation:

(5) $$\Delta G_{formation}=\gamma \Delta A+T\frac{nk}{\emptyset }\left(\emptyset ln\emptyset +\left(1-\emptyset \right)\ln\left(1-\emptyset \right)\right)$$

In a given system, k, T, ∅ are constant parameters. Reducing the particle size will increase the surface area of the interface between oil and water phases (ΔA) and the number of particles (n) and also affect the interfacial tension (γ). Among these parameters, since the interfacial tension relies on the curvature of a surfactant monolayer, the influence of droplet size on the interfacial tension is more complex. The interfacial tension of the droplet can be modified using equation [33]:

(6) $$\gamma =\gamma_{0}+\left(\gamma_{\infty }-\gamma_{0}\right)\frac{{\left(R_{0}-R\right)}^{2}}{R_{0}^{2}-R^{2}}$$

where $\gamma_{0}$ is the interfacial tension when the surfactant monolayer is at its optimum curvature with the radius of $R_{0}$, $\gamma_{\infty }$ is the interfacial tension at a planar oil–water interface, R is the particle radius. In this model, the interfacial tension was minimized with the appropriate particle radius. As a result, the free energy profile can reach a global or local minimum position. Assuming SEDDSs spontaneously form droplets with the optimized radius, the free energy profiles of $\Delta G_{formation}$ of these SEDDS were plotted according to Equations (5) and (6).

### 2.7. Caco-2 Cell Lines Culture and Permeability Studies

Human colorectal adenocarcinoma colon epithelial cells (Caco-2) were purchased from the Type Culture Collection of Chinese Academy of Sciences (Shanghai, China). Caco-2 cells were cultured in a growth medium consisting of Dulbecco’s modified Eagle’s medium (DMEM; Corning, Shanghai, China) supplemented with 20% fetal bovine serum (FBS; Corning, China) and antibiotics (antibiotic−antimycotic solution; Corning, China). Cells were cultured in a cell incubator at 37 °C with 5% CO_2_.

Caco-2 cells were grown and harvested for seeding into the basal compartment of the Transwell insert (Nunc, Thermo, Waltham, MA, USA) at a density of 5 × 10^4^ cells/mL. After 21 days of culture, Caco-2 cells were fully differentiated and exhibited similar morphology and functionality to small intestinal epithelial cells. The transepithelial electrical resistance (TEER) of the cell monolayer was measured using a RE1600 (Beijing Metalworking Hongtai Technology Co., Ltd., Beijing, China). Compartments with a TEER value exceeding 500 Ω·cm^2^ were selected for the transport study.

A total of 0.5 mL of test samples and 0.6 mL of HBSS were added to the apical and basolateral sides, respectively, for the transport studies. At 0.5 h, 1 h, 2 h, and 4 h, 100 μL samples were collected from the basolateral side, and the chambers were replenished using 100 μL fresh pre-warmed HBSS immediately.

The collected solutions were diluted two-fold with methanol, centrifuged, and then analyzed by HPLC (Section 2.3.1). The apparent permeability coefficient (*P*_app_, cm·s^−1^) was calculated using the following equation.

(7) $$P_{app}=\frac{dQ}{dt}\times \frac{1}{{AC}_{0}}$$

where $\frac{dQ}{dt}\;$is the drug permeation rate ($\mu g\cdot S^{-1}$), A is the surface area of the insert membrane (0.43 cm^2^), and $C_{0}$ is the initial concentration of progesterone in the donor solution ($\mu g\cdot {\mathrm{mL}}^{-1}$).

### 2.8. Drug Absorption Approach Study

#### 2.8.1. Pharmacokinetic Study in ICR Mice

Male ICR mice (30 ± 2 g) were fasted overnight and randomly divided into four groups: A, B, C, and D (n = 6). Group A and group B were treated with Utrogestan^®^ (Cyndea Pharma, S.L., Soria, Spain) at a dose of 200 mg/kg by oral gavage. Group C and Group D were orally administered with MCT&LCT-SEDDS at an equivalent dose of 200 mg/kg progesterone. After oral administration, a blood sample (50 μL) was collected from eye ground vein at 0.5, 1.5, 4, and 12 h for group A and group C; 1, 2, 8, and 24 h for group B and group D. Each sample was immediately transferred into Lithium Heparin tubes and centrifuged at 5000 rpm for 10 min. A total of 100 μL supernatant was collected and stored at −20 °C. The drug concentrations in plasma were determined using the HPLC method described in Section 2.3.2.

Pharmacokinetic parameters were calculated using DRUG AND STATISTICS software (DAS, Version 2.0; Mathematical Pharmacology Professional Committee of China, Shanghai, China).

#### 2.8.2. Chylomicron Blocking Model Study

Male ICR mice (30 ± 2 g) were fasted overnight and randomly divided into two groups: E and F (n = 6). All mice were pretreated with cycloheximide by injection intraperitoneally. After 1 h, group E and group F were orally administered MCT&LCT-SEDDS at an equivalent dose of 200 mg/kg progesterone. After oral administration, a blood sample (100 μL) was collected from the eye ground vein at 0.5, 1.5, 4, and 12 h for group E, 1, 2, 8, and 24 h for group F. The drug concentrations in plasma were determined using the HPLC method as described in Section 2.3.2.

### 2.9. Statistical Analysis

All data are presented as mean ± standard deviation (SD) of more than three experiments or samples. A two-tailed unpaired Student’s *t*-test was used for statistical analysis. *p* < 0.05 was considered statistically significant

## 3. Results and Discussion

### 3.1. Solubility Study

**Figure 1:**
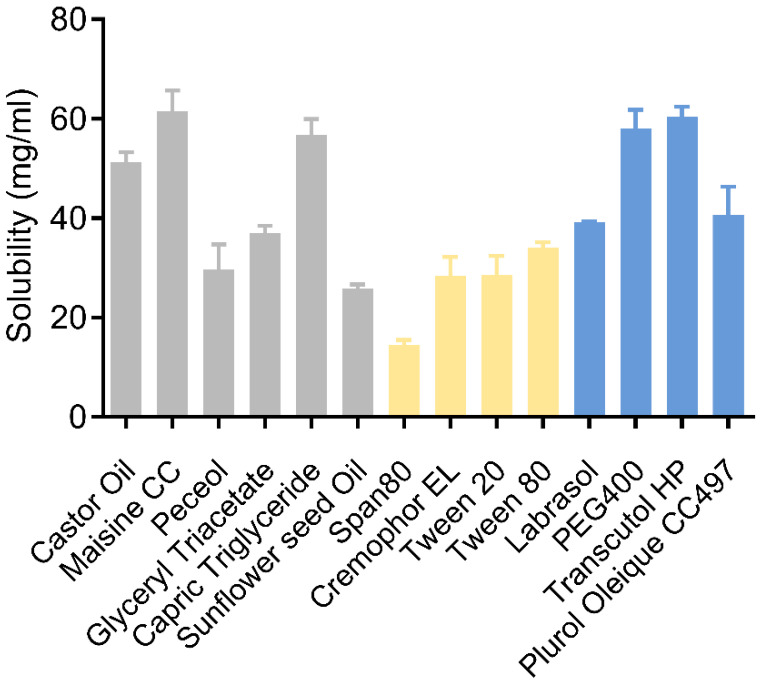
The equilibrium solubility of progesterone in various vehicles (oils shown in gray bars, surfactants shown in yellow bars, and co-surfactants shown in blue bars) at 25 °C.

Solubility of the drug in oil, surfactant, and/or co-surfactant is one of the primary parameters commonly evaluated in the design and development of SEDDS. The drug solubility in these excipients should be adequate to promote the drug loading of the product. In the screening of the oil phase, progesterone was found to have the highest solubility in Maisine CC (61.46 mg/g) and caprylic/capric triglyceride (56.74 mg/g), where the former is one of the LCT and the latter belongs to MCT (Figure 1, gray bars).

Three non-ionic surfactants were evaluated in this work, considering the adverse effect of ionic surfactants in the gastrointestinal (GI) tract (Figure 1, yellow bars). The solubility of progesterone in Tween 80 was determined to be 34.14 mg/g, higher than that in Tween 20 (28.52 mg/g) and Cremophor EL (28.40 mg/g). The HLB value of the surfactant is another factor that may affect the emulsion of the system [35]. This factor was neglected in this screening because the HLB value of Tween 80 is similar to those of Tween 20 and Cremophor EL (15, 16.7, and 16, respectively).

Co-surfactant was introduced into the SEDDS to facilitate the formation of the micro- or nano-emulsion system and improve the drug loading of the product. Ethanol, propylene glycol, and other solvents were typically used as co-surfactants. However, a high proportion of the organic solvent may be absorbed by the gelatin shell of the product and lead to the softening and deformation of the capsule. Therefore, several non-alcoholic co-surfactants have been screened in this work (Figure 1, blue bars). The solubility of progesterone in Transcutol HP was detected to be 60.34 mg/g, higher than those in Labrasol, PEG 400, Plurol Oleique CC 497, and Span 80. Thus, according to solubility data, Maisine CC, capric triglyceride, Tween 80, and Transcutol HP were selected for the construction of ternary phase diagrams to develop progesterone-loaded SEDDS.

### 3.2. Pseudo-Ternary Phase Diagram Construction

**Figure 2:**
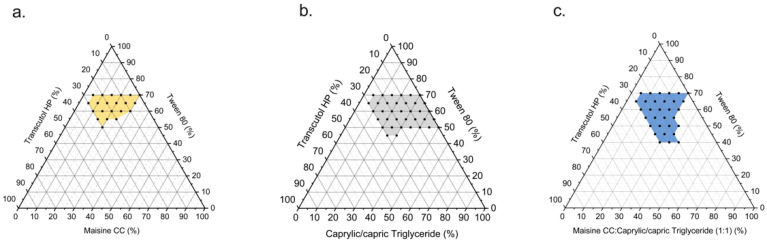
Ternary phase diagrams for surfactant (Tween 80), co-surfactant (Transcutol HP), and different oils: (**a**) Maisine CC, (**b**) capric triglyceride, and (**c**) 1:1 ratios of mixed oil (Maisine CC and capric triglyceride) shown in weight fractions.

Pseudo-ternary phase diagrams were constructed to plot the self-emulsifying area of SEDDS, using the oil (Maisine CC, capric triglyceride, or the mixture), surfactant (Tween 80), and co-surfactant (Transcutol HP) phases. The boundary of the ternary phase diagram (Figure 2) was established based on the identification of the transparent and turbid solutions.

The emulsion range of MCT SEDDS (Figure 2b, gray area) was larger than that of the LCT SEDDS (Figure 2a, yellow area). The results may be attributed to the fact that MCT is a more appropriate oil phase for the generation of self-emulsifying [36]. With the mixing of MCT and LCT, the region of the transparent solution (Figure 2c, blue area) was further increased, indicating that the mixture oil facilitated the emulsification of the system.

Particularly, less surfactant is demanded to emulsify the oil phase in the MCT&LCT mixture system. For instance, only 40% *w*/*w* Tween 80 is required to emulsify 40% *w*/*w* oil phase with the presence of 20% *w*/*w* Transcutol in the MCT&LCT SEDDS. However, the weight fraction of Tween 80 should be improved to 50% *w*/*w* to form a semi-transparent solution in the MCT system. In the LCT system, no transparent solution could be observed when the oil phase was set at 40% *w*/*w*.

### 3.3. Droplet Size and Zeta Potential Analysis

**Figure 3:**
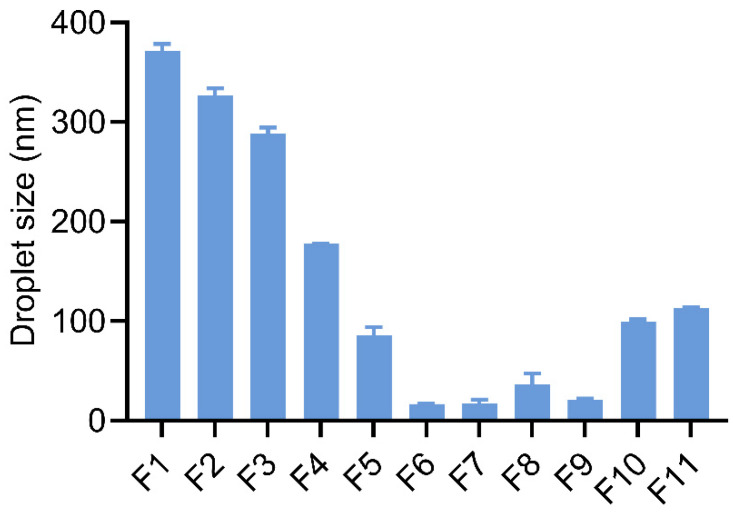
The droplet size of SEDDSs with different MCT/LCT ratios (formulation composition see Table 2).

Droplet size distribution is a critical factor in the evaluation of self-emulsifying performance. Smaller sizes provide a larger surface area between the emulsion droplets and the aqueous medium, which can enhance the rate and degree of drug release and absorption [37]. The particle size of the LCT-SEDDS formulation (F1) was determined to be 371.60 ± 6.90 nm. MCT-SEDDS (F11) exhibited a smaller particle size, detected to be 113.50.41 ± 0.34 nm. It is noteworthy to see the emulsion droplet sizes were significantly reduced by changing the MCT/LCT ratio (Figure 3) without altering the surfactant and co-surfactant composition. The optimized droplet size was 21.23 ± 0.30 nm, in which the LCT/MCT ratio was set to be 5:5 (F6). In terms of particle dispersion, the PDIs of F1, F6, and F11 systems were detected to be 0.29 ± 0.015, 0.37 ± 0.016, and 0.28 ± 0.007, indicating a uniform distribution of these droplets (Table 3).

Furthermore, particle zeta potential is another important parameter that may reflect the stability of the emulsion, influence the interaction between the particle and mucin layer, and affect the subsequent drug absorption in the GI tract [38,39,40]. The particle zeta potential is reasonably considered to rely on the surfactant and co-surfactant since those are condensed on the surface of the emulsion particle. However, in this work, the type of oil phase was found to change the particle surface zeta potential. The zeta potential of LCT SEDDS (F1) was detected to be a positive value, +4.35 ± 0.33 mV. The data of the MCT SEDDS (F11) changed to a negative value, −5.49 ± 0.15 mV. By simply mixing the MCT and LCT oil phase with the weight ratio of 1:1, the absolute value of zeta potential was significantly different from the other two samples (*p* < 0.01), determined to be −7.28 ± 0.73 mV. However, the absolute value of zeta potentials may not be sufficient to repulse the particle and distinguish the stability of MCT, LCT, and MCT&LCT SEDDS.

### 3.4. Particle Radius Influence on the Thermodynamic Profiles of SEDDSs

It was widely revealed that particle radius is an important parameter that influences the system’s physical stability. This manuscript found that the droplet size was significantly reduced to around 20 nm by mixing the MCT and LCT as a homogeneous oil phase. A simple thermodynamic model was employed to understand the influence of size distribution on the system’s free energy profiles and its physical stability.

**Figure 4:**
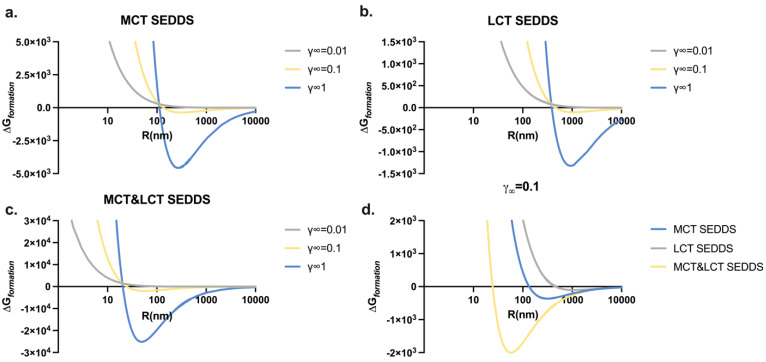
Schematic Gibbs free energy ($\Delta G_{formation}$) profiles as a function of droplet radius of (**a**) MCT SEDDS, (**b**) LCT SEDDS, and (**c**) MCT&LCT SEDDS with $\gamma_{\infty }=0.01,\;0.1\;\mathrm{or}\;1$; (**d**) MCT, LCT, and MCT&LCT SEDDSs with $\gamma_{\infty }=0.1$.

The particle sizes of spontaneously formed droplets determined using the DLS were used to plot the Gibbs free energy ($\Delta G_{formation}$) profiles of MTC, LCT, and MCT&LCT SEDDSs (Figure 4). For a typical SEDDS, the lipophilic phase could be well-dispersed in the aqueous environment with the external mechanical energy of mixing or stirring. Nevertheless, the $\Delta G_{formation}$ sharply increased when reducing the droplet radius, which disfavored the generation of colloids. In some cases, a global or local $\Delta G_{formation}$ minimum position could be reached with an appropriate value of $\gamma_{\infty }$ and R (yellow and blue curves in Figure 4a–c). This position with a negative $\Delta G_{formation}$ leads the system to reach a stable or meta-stable state. Any change in the particle size may move the system away from this position, increase the value of $\Delta G_{formation}$ and, therefore, drive the particle split or aggregate until the optimum size.

When setting the value of $\gamma_{\infty }$ at 0.1, the droplet radius R of the MCT&LCT SEDDS at the minimum position is much smaller than the MCT and LCT SEDDSs (Figure 4d). In addition, the blended MCT&LCT system achieves a deeper free energy minimum position with a more negative value of $\Delta G_{formation}$, compared to the MCT and LCT. According to Equations (5) and (6), these thermodynamic advantages of the blended system may be attributed to the fact that the complementary of MCT and LCT in the generation of the oil phase influences the interfacial tension of the droplet and, thus, the value of $\Delta G_{formation}$. On the other hand, the mixture of MCT and LCT could reduce the value of $\Delta G_{formation}$ through governing the configuration entropy of the colloid formation.

### 3.5. In Vitro Lipolysis Study

In the GI lumen, lipids are digested by lipase and colipase to form free acids, monoglycerides, and diglycerides. Along with lipid digestion, drugs in the SEDDS formulation form complexes consisting of bile salts, lecithin, chylomicron, and other components. These micelles, colloids, and other combinations will affect the drug distribution and, therefore, the bioavailability. The in vitro lipolysis study is a useful tool to predict the food effect of poorly water-soluble drugs [31,41]. Three phases could be observed after the lipolysis study followed by ultracentrifugation, named oil phase, aqueous phase, and pellet phases, from top to bottom. The oil phase was composed of the undigested lipophilic substance with low density. The pellet phase was formed due to the aggregation and precipitation of the undissolved drugs. In general, drugs that dispersed in the aqueous phase were easier to be absorbed than those in other layers.

**Figure 5:**
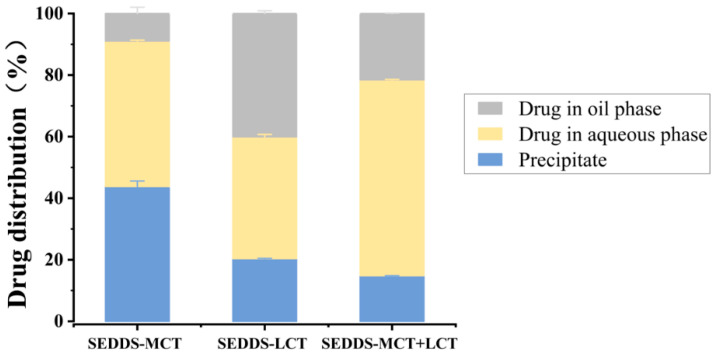
Distribution of Progesterone into the oil, aqueous, and pellet phases in in vitro lipolysis study, data shown in weight fractions.

MCT&LCT SEDDS formulation showed the highest drug content in the aqueous phase after the lipolysis study (Figure 5). In this system, 63.62% *w*/*w* of drugs were dispersed in the aqueous phase, suggesting the MCT&LCT system has a better solubility improvement of progesterone than the individual oil system. MCT SEDDS formulation has the highest progesterone concentration in the pellet phase (43.83% *w*/*w*). It was greater than the LCT SEDDS (20.37% *w*/*w*) and MCT&LCT SEDDS (14.82% *w*/*w*) system. The results suggest that the mixed oil system could prevent the drug from aggregating or precipitation during digestion.

### 3.6. Permeability of Progesterone

Although a number of strategies could improve the solubility of hydrophobic drugs, drug absorption may not be enhanced since the solubilization techniques may undermine the drug permeability [42,43,44]. To investigate the influence of size-reduced MCT&LCT on drug permeability, the Caco-2 cell model was employed. Caco-2 cells are a typical model to evaluate the human intestinal absorption of drugs and other substances [45]. They exhibit some morphology and characteristic similarities to the human intestinal epithelium, including microvilli structure, tight junctions between cells, secretion of hydrolases, and expression of P-glycoprotein et al. [46,47].

**Figure 6:**
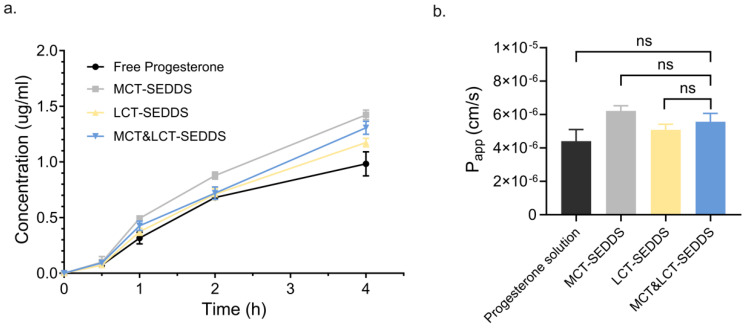
(**a**) Cumulative transport rate (%) of the progesterone in Caco-2 models using free progesterone solution, MCT-SEDDS, LCT-SEDDS, and MCT&LCT-SEDDS formulations as a function of time (h); (**b**) $P_{app}$ of progesterone determined using those formulations (ns represents no significant difference).

The $P_{app}$ of pure progesterone solution is higher than $1\times 10^{-6}\;\mathrm{cm}/s$, which suggests that it could be classified as a high-permeability substance in the Caco-2 model (Figure 6a). Therefore, permeability is not the limitation step of the absorption of progesterone. In the cases of SEDDSs, three formulations with various oil phases could not change the permeability of the drug significantly (*p* > 0.05, Figure 6b). This result demonstrated that the SEDDS formulation may not regulate the permeability of progesterone across the epithelium cells, regardless of the mixed oil phase leading to particle size, zeta potential, and solubility changing. The SEDDS influence on the P-gp has not been investigated in this work since the model drug progesterone is not the typical substrate of the P-gp.

### 3.7. Pharmacokinetics Study

Progesterone is an endogenous steroid hormone that plays an important role in the hormonal therapy of pregnancy. It was approved by the FDA for use in the supplementation of the luteal phase and maintenance of early pregnancy as part of an Assisted Reproductive Technology (ART) [48]. However, progesterone has a limited bioavailability due to its poorly water solubility. Clinically, progesterone is administrated in oil intramuscular, intravaginal, or oral. Oil intramuscular is one of the most common ways to administrate progesterone with high bioavailability but low price. However, the oil matrix of the formulation leads to pain, itching, irritation, and/or swelling at the injection site [49]. Oral administration is the most commonly used drug delivery system with high patient compliance. Utrogestan^®^ is an oral administrated commercial product combating this problem by dispersion of the progesterone microcrystals into an oily matrix.

**Figure 7:**
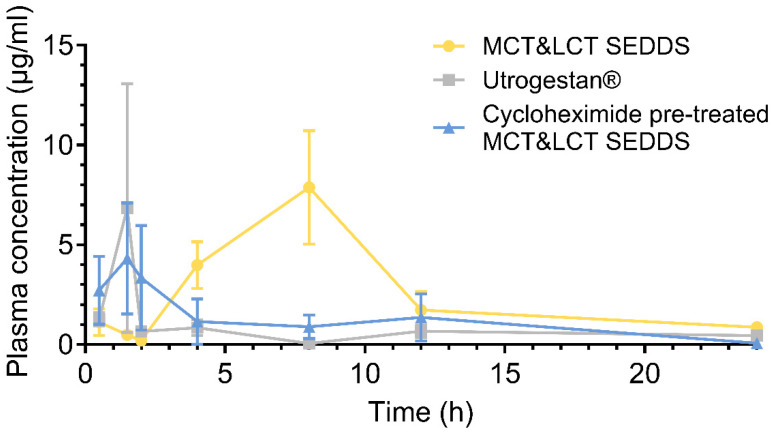
Concentration-time profiles of progesterone in plasma after oral administration of MCT&LCT-SEDDS, Utrogestan^®^, and cycloheximide pre-treated MCT&LCT-SEDDS in mice (n = 6).

SEDDS is a lipid-based strategy that increases the drug oral absorption by forming fine dispersed drug-contained emulsion droplets. It is absorbed by the GI tract through the transcellular, paracellular, or lymphatic circulation approach [17,50]. The drug absorption of Utrogestan^®^ and MCT&LCT-SEDDS was investigated using the ICR mouse model. The results suggested that MCT&LCT-SEDDS has a significantly higher drug absorption than Utrogestan^®^ (Figure 7, Table 4, *p* < 0.01). The relative bioavailability of the drug in the MCT&LCT-SEDDS group was 3.82 times higher than the Utrogestan^®^ group. The MCT&LCT SEDDS improved the relative bioavailability of the progesterone was achieved by prolonging the $T_{max}$ of progesterone concentration without changing the $C_{max}$. In detail, the group administrated by Utrogestan^®^ has a $C_{max}$ of 8.14 ± 14.60 μg/mL, the SEDDS group has a similar $C_{max}$ of 8.58 ± 6.34 μg/mL. The $T_{max}$ of the drug absorption was delayed from 1.33 ± 1.37 h by administrated of Utrogestan^®^ to 6.08 ± 3.17 h by administrated of SEDDS.

Additionally, the serious liver first-pass effect is another critical parameter that leads to the low bioavailability of progesterone. In particular, the metabolite substance allopregnanolone was revealed, which refers to the side effects such as dizziness, sedation, and headache of oral administration of progesterone [51]. SEDDS is a promising way to reduce the liver first-pass effect. This is due to the fact the triglycerides in the formulation could be absorbed through the lymphatic pathway with the formation of chylomicron, which consists primarily of triglyceride core, esterified cholesterol, apoproteins, and phospholipids [6,7,26,27,28,29,30]. The drug-associated chylomicron is an ideal vehicle to transport the hydrophobic drug into the system circulation through the lymphatic pathway, thereby bypassing first-pass metabolism.

The cycloheximide model was commonly applied to qualitatively study the drug transport mechanisms, although some of the literature suggested that it may overestimate the lymphatic transport of some drugs [52]. In this work, for the mice pretreated with cycloheximide, a substance that blocks the chylomicron flow, the benefit of SEDDS in cases of delivering progesterone was reduced. The $AUC_{0-t}$ of the cycloheximide pretreated SEDDS group was reduced to 23.72 ± 13.61 μg/mL·h. The relative bioavailability of the cycloheximide pretreated SEDDS group was 35.76% compared with the SEDDS. These results demonstrated that the SEDDS may improve the progesterone through the lymphatic pathway in the mouse model.

## 4. Conclusions

A SEDDS with reduced droplet size was designed using the mixed MCT and LCT. By blending these excipients, the MCT&LCT SEDDS had a larger self-emulsifying range than the MCT and LCT SEDDS. Less amount of surfactant is required to retain the transparent emulsion in the MCT&LCT system, suggesting a better self-emulsifying ability. The development of the novel SEDDS provides a prospect of oral administration of progesterone with high stability and improved bioavailability.

## Figures and Tables

**Table 1:** Composition of SEDDS formulations.

Composition	MCT-SEDDS	LCT-SEDDS	MCT&LCT-SEDDS
Progesterone (g)	1.0	1.0	1.0
Maisine CC (g)	0	10.0	5.0
Capric Triglyceride (g)	10.0	0	5.0
Tween-80 (g)	27.5	27.5	27.5
Transcutol HP (g)	12.5	12.5	12.5

**Table 2:** Composition of SEDDSs with different MCT/LCT ratios.

Composition (Weight Fractions %)	Formulation										
	F1	F2	F3	F4	F5	F6	F7	F8	F9	F10	F11
Maisine CC	20	18	16	14	12	10	8	6	4	2	0
Capric Triglyceride	0	2	4	6	8	10	12	14	16	18	20
Tween-80	55	55	55	55	55	55	55	55	55	55	55
Transcutol HP	25	25	25	25	25	25	25	25	25	25	25
MCT/LCT ratio	0:10	1:9	2:8	3:7	4:6	5:5	6:4	7:3	8:2	9:1	10:0

**Table 3:** Droplets size and Zeta potential of SEDDS formulations in dispersion test.

Formulation	Droplet Size (nm)	PDI	Zeta Potential (mV)
F1 (LCT SEDDS)	371.60 ± 6.90 **	0.29 ± 0.015	+4.35 ± 0.33
F6 (MCT&LCT SEDDS)	21.23 ± 0.30	0.37 ± 0.016	−7.28 ± 0.73
F11 (MCT SEDDS)	113.50 ± 0.34 **	0.28 ± 0.007	−5.49 ± 0.15

** represents *p* < 0.01, data compared with F6.

**Table 4:** Main pharmacokinetic parameters of progesterone after oral administration of Utrogestan^®^ and MCT&LCT SEDDS (with and without chylomicron flow blocking using cycloheximide) in mice.

Pharmacokinetics Parameters	Utrogestan^®^	MCT&LCT-SEDDS	Cycloheximide+ MCT&LCT-SEDDS
$C_{max}\;\left(\mu g/\mathrm{mL}\right)$	8.14 ± 14.60	8.58 ± 6.34	9.19 ± 6.26
$T_{max}\;\left(h\right)$	1.33 ± 1.37	6.08 ± 3.17 **	3.58 ± 3.46
$t_{1/2}\;\left(h\right)$	6.69 ± 5.37	6.84 ± 5.08	3.72 ± 2.84
$AUC_{0-t}\;\left(\mu g/\mathrm{mL}\cdot h\right)$	17.34 ± 8.58	66.33 ± 37.22 *	23.72 ± 13.61 ^#^
$AUC_{0-\infty }\;\left(\mu g/\mathrm{mL}\cdot h\right)$	21.71 ± 13.58	74.89 ± 35.12 **	26.56 ± 15.43 ^#^
$MRT_{0-t}\;\left(h\right)$	9.04 ± 5.02	9.16 ± 1.78	5.00 ± 2.47 ^##^
$CL_{z}\;\left(\frac{L}{h}/\mathrm{kg}\right)$	1.27 ± 0.71	0.34 ± 0.19 *	1.06 ± 0.71 ^#^
Relative $AUC_{0-t}$ (compare to Utrogestan^®^)	-	382.52%	136.79%
Relative $AUC_{0-t}$ (compare to MCT&LCT-SEDDS)	-	-	35.76%

Data were analyzed using the software DAS 2.0. * and ** represent *p* < 0.05 and *p* < 0.01, data compared with Utrogestan^®^. ^#^ and ^##^ represent *p* < 0.01 and *p* < 0.01, data compared with MCT&LCT-SEDDS.

## Data Availability

Data are unavailable due to privacy restrictions.

## Abbreviations

The following abbreviations are used in this manuscript:

FDA	Food and Drug Administration
HPLC	High-performance liquid chromatography
LCT	Long-chain length triglycerides
MCT	Medium-chain length triglycerides
SEDDS	Self-emulsifying drug delivery system
SOR	Surfactant-to-oil ratio
